# Long and oriented graphene nanoribbon synthesis from well-ordered 10,10′-dibromo-9,9′-bianthracene monolayer on crystalline Au surfaces†

**DOI:** 10.1039/d2ra07570a

**Published:** 2023-05-09

**Authors:** Masahiro Yano, Satoshi Yasuda, Katsuyuki Fukutani, Hidehito Asaoka

**Affiliations:** a Research Group for Surface and Interface Science, Advanced Science Research Center, Japan Atomic Energy Agency 2-4 Shirakata Tokai Ibaraki 319-1195 Japan yano.masahiro@jaea.go.jp asaoka.hidehito@jaea.go.jp; b Institute of Industrial Science, The University of Tokyo 4-6-1 Komaba, Meguro-ku Tokyo 153-8505 Japan

## Abstract

Bottom-up synthesis on metal surfaces has attracted attention for the fabrication of graphene nanoribbons (GNRs) with atomically-precise chemical structures to realize novel electronic devices. However, control of length and orientation on surfaces during GNR synthesis is difficult, thus, achieving longer and aligned GNR growth is a significant challenge. Herein, we report GNR synthesis from a well-ordered dense monolayer on Au crystalline surfaces for long and oriented GNR growth. Scanning tunneling microscopy showed that 10,10′-dibromo-9,9′-bianthracene (DBBA) precursors deposited on Au(111) at room temperature self-assembled into a well-ordered dense monolayer, and the straight molecular wire structure was formed where Br atoms in each precursor were adjacent along the wire axis. The DBBAs in the monolayer were found to be hardly desorbed from the surface under subsequent heating and efficiently polymerize along with the molecular arrangement, resulting in more long and oriented GNR growth compared to the conventional growth method. The result is attributed to be suppression of random diffusion and desorption of the DBBAs on the Au surface during polymerization due to the densely-packed DBBA structure. Additionally, an investigation of the effect of the Au crystalline plane on the GNR growth revealed further anisotropic GNR growth on Au(100) compared to Au(111) due to the stronger interactions of DBBA with Au(100). These findings provide fundamental knowledge for controlling GNR growth from a well-ordered precursor monolayer to achieve more long and oriented GNRs.

## Abstract 2

The length and orientation of graphene nanoribbons (GNRs) synthesized from molecules fixed in monolayers are significantly different from those synthesized from diffusing molecules.

## Graphical abstract

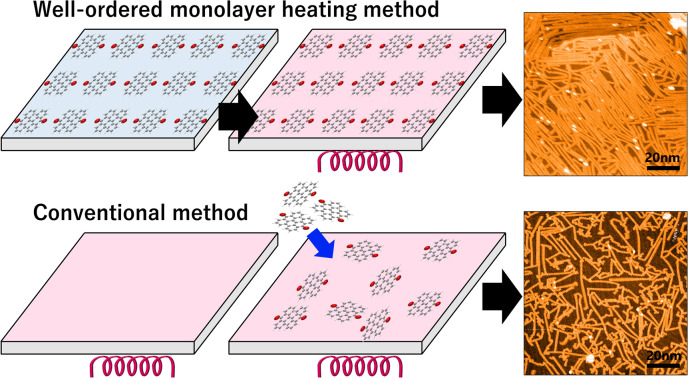

## Introduction

Graphene has attracted the interest of researchers worldwide due to its unique characteristics.^1–5^ In particular, carrier mobility is more than 100 times that of Si, presumably making it an excellent choice for the development of high-speed field-effect transistors (FETs).^1,2^ Despite the excellent properties, the absence of a sizable bandgap makes limitations for application of graphene in FETs.^6,7^ Graphene nanoribbon (GNR), a quasi-one-dimensional graphene structure,^8^ is promising for FET applications due to the controllability of its electronic structure, including the bandgap, by its width and edge structure.^9^ Electron or helium ion beam lithography is a top-down technique for producing GNR from graphene and has been used to fabricate GNRs with widths of ∼10 nm, which is sufficient for the formation of a sizable bandgap in room temperature applications.^10–12^ Although the top-down process can produce GNR at precise positioning on substrates, it sometimes causes damage to the GNR and makes the GNR edge structure rough; thus, precise control of the electronic structure is difficult.^10,13–16^

In 2010, Cai and coworkers brilliantly solved this difficulty by employing a bottom-up synthesis approach.^17^ The process is based on chemical vapor deposition (CVD), and the GNRs with a uniform and precise edge structure could be successfully fabricated on the metal surface. 10,10′-dibromo-9,9′-bianthracene (DBBA), a brominated aromatic hydrocarbon substituted at both ends, was used as a building block precursor, and sublimation of DBBA on the Au(111) surface maintained at ∼200 °C induced dehalogenation and radicals in the precursors on the surface. The adsorbed and diffused radical precursors were polymerized on the surface as oligomers by covalent C–C bond formation (surface-assisted Ullman coupling reaction). Subsequent heating to ∼400 °C could cyclodehydrogenate the polymers allowing seven-carbon-atom-wide armchair GNR (7-aGNR) formation.

However, controlling the GNR length and the orientation on the surface remains a difficulty with the bottom-up CVD synthesis approach. The method reported in the early stages involved simultaneous deposition of precursors and polymerization by heating.^17^ Later, depositing precursor molecules on a substrate with low coverage followed by heating was also reported.^18–24^ However, because of low adsorption coverage less than one monolayer (ML)^18–22^ as well as consequent desorption of the precursors by heating,^23,24^ the precursors in both methods tend to polymerize by free diffusion on the surface, causing the formation of randomly oriented oligomers. Furthermore, the collision between the oligomers during the polymerization process results in the formation of short and randomly oriented GNRs. For the development of GNR-based FETs, the short and randomly oriented GNRs would limit the charge transport property due to the hopping of charge carriers between GNRs, reducing the speed of the FETs.^25^ Thus, the production of longer and unidirectional GNRs through bottom-up synthesis remains a great challenge for the realization of high-performance GNR-based FETs.

For this reason, various bottom-up processes have been developed to achieve long and unidirectional GNR growth by utilizing the surface step structure of vicinal surfaces,^26–28^ and various shaped molecular building blocks.^29,30^ However, these methods require precise and complicated design of the precursor shape and surface structures, resulting in time and effort consumption. As an alternative method, GNR growth from well-ordered precursor monolayers has attracted attention^31^ because the monolayer formation would be able to suppress the molecular desorption and precursor diffusion on the surface by intermolecular interaction during heating, expecting long and oriented GNR growth. In addition, it is not necessary to utilize the specific surface structure and precursor molecules. Practically, well-ordered 4,4′′-dibromo-*p*-terphenyl (DBTP) molecules consisting of linearly connected three benzene rings could successfully polymerize by heating with less desorption and diffusion of the precursors, and consequent long and oriented GNR growth was achieved.^31^ The study clearly demonstrated that the GNR growth technique from the well-ordered monolayer is one of the promising candidates for long and unidirectional GNR growth.

In general, to effectively utilize characteristics of the growth technique, it is important to consider parameters affecting the GNR growth, such as precursor shape and surface structure. In fact, the DBTP used in the previous study seems to be inappropriate as the precursor for the technique because of difficulty of uniform GNR formation. The DBTPs self-assemble into linear molecular wire structures on Au(111), but the lateral molecular wires are needed to polymerize for GNR formation. Since the lateral fusion process randomly occurs, the formation of branched GNRs with various widths and lengths is inevitable despite long and oriented GNR growth. Hence, it is suitable to adopt precursors which do not involve the complicated lateral fusion processes during GNR formation. However, there is still no study on the GNR growth from the monolayer using the related precursors.

In this study, as the precursor, the DBBA molecule was employed for the well-ordered precursor monolayer technique. As already described, the DBBA is known to not only form well-ordered dense monolayer but also produce only 7-aGNR without lateral fusion process. To obtain further fundamental knowledge, surface structure effect was also verified. Au(100) was also chosen as the surface to support anisotropic and long GNR growth because previous literature showed a relatively strong interaction of Au(100) with organic molecules compared to Au(111).

Scanning tunneling microscopy (STM) demonstrated that deposition of 1 ML of DBBA at room temperature produced a well-ordered dense monolayer on Au(111) as expected, in which DBBAs were ordered in a straight line, and the Br atoms in each molecule were oriented along the 〈110〉 direction on Au(111). We also confirmed that the desorption, random diffusion, and rotation of DBBAs in the monolayer by subsequent heating were suppressed due to the intermolecular interaction in the densely packing. The resultant monolayer efficiently induced polymerization of covalent C–C intermolecular connections between the ordered precursors by a debromination reaction, achieving longer and anisotropic GNR growth compared to the CVD method. The study of Au crystalline plane on the growth also showed further anisotropic growth on Au(100) compared to Au(111), but there was few effect for longer growth, probably owing to the strong interaction of the precursors with Au(100). These findings are helpful as an insight to design procedures for the more long and oriented GNRs synthesis from the well-ordered precursor monolayer for the development of GNR-based applications.

## Experimental

Au(111) and Au(100) surfaces were used as Au crystalline surface templates and were prepared using conventional cycles of Ar^+^ ion sputtering (1.5 keV) with subsequent annealing at approximately 600 °C in an ultra-high vacuum (UHV). DBBA (Tokyo Chemical Industry Co., Ltd, 98, +%) was used without further purification.

In this study, two different GNR synthesis methods were performed to verify the effect of the synthetic methodology on the orientation and length of GNRs. The first method was the monolayer heating method, in which GNR was grown from the as-grown well-ordered precursor monolayer. The well-ordered dense DBBA monolayer (∼1 ML) was prepared by depositing DBBA molecules on the Au surface from a homemade Knudsen cell at a rate of ∼0.05 ML min^−1^ while maintaining the substrate at room temperature (RT). The DBBA deposition rate was monitored *in situ* using a quartz crystal microbalance. The molecular arrangements were then evaluated using UHV STM (JSPM-4500A, JEOL Japan) operating in constant current mode with mechanically cut W tips at RT under 5 × 10^−8^ Pa. Next, the monolayers were heated at 200 °C for 30 minutes to polymerize the molecules by a debromination reaction, and then at 400 °C for 30 minutes to cyclodehydrogenate the polymers. The monolayers were heated by ceramic heaters in contact with the back of the Au substrate, and the surface temperature of the substrate was monitored by a radiation thermometer and increased at a rate of 1 °C min^−1^. The produced GNR structure was analyzed by STM. All operations were carried out in the UHV chamber without exposure to the atmosphere.

The second method was the well-known CVD based on simultaneous DBBA deposition and polymerization on the heated Au surface, which is a model for GNR synthesis from randomly diffusing precursor molecules, as reported by Cai *et al.*^17^ Briefly, after simultaneous DBBA deposition and polymerization at 200 °C for 30 minutes, GNR was synthesized by a cyclodehydrogenation reaction at 400 °C for 30 minutes, and then STM was performed for the structural analysis. After STM analysis, the Raman spectroscopy (DXR Microscope, Thermo Fisher Scientific) was performed in the air to characterize the produced GNRs with a 532 nm laser excitation source at 1 mW.

## Results and discussion

### GNR growth on Au(111) by monolayer heating and CVD methods

**Fig. 1:**
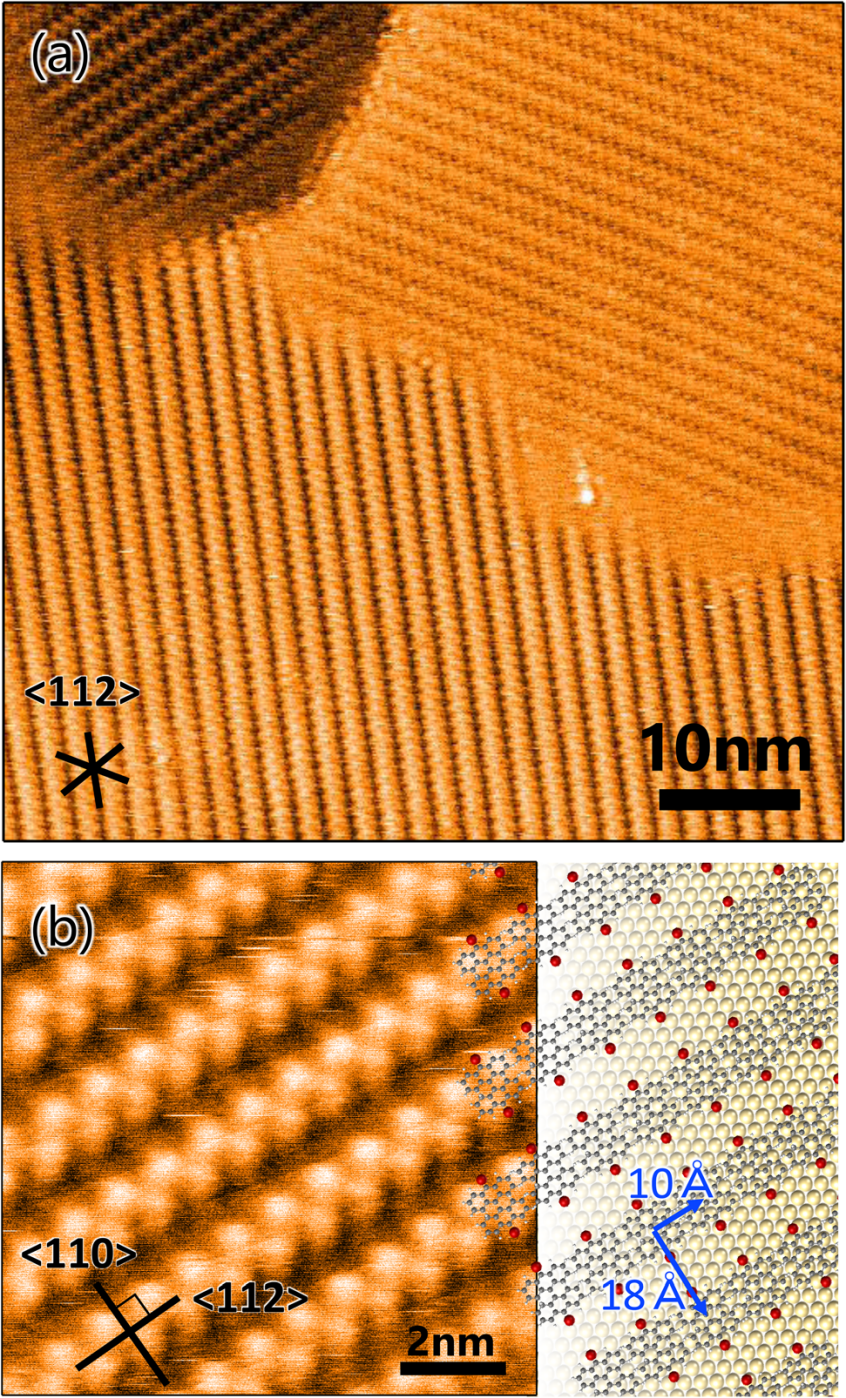
(a) STM image of 10,10′-dibromo-9,9′-bianthracenes monolayer on Au(111) (*V*_S_ = 2.0 V, *I*_T_ = 50 pA). (b) Enlarged STM image (*V*_S_ = 2.0 V, *I*_T_ = 90 pA) with molecular arrangement model expected from unit cell size (10 Å × 18 Å). The grey, red, and yellow balls represent carbon, bromine, and gold atoms, respectively. Inserted lines represent the 〈112〉 and 〈110〉 directions of the substrate.

A well-ordered dense DBBA monolayer prepared by deposition of DBBA on clean Au(111) was first characterized by STM. The STM image of the DBBA monolayer (Fig. 1a) showed well-ordered close-packed chain-like structures parallel to the 〈112〉 direction. There are three oriented domains reflecting a sixfold symmetric atomic arrangement of the substrate. The domains grew on the Au(111)-herringbone reconstruction (see Fig. S1 in ESI†) to 30 nm to 100 nm, which is longer than the herringbone bending period, suggesting that undulation of the reconstructed Au(111) has few effects on the DBBA monolayer formation. The enlarged STM image of the DBBA monolayer (Fig. 1b) showed dumbbell-like protrusions representing up-tilted phenyl groups in the molecules. Each chain-like array running from right to left corresponded to a molecular array adjacent to the phenyl groups, and each Br atom (red balls in Fig. 1b) in the DBBAs was oriented in the 〈110〉 direction parallel to the Br–Br axis. The image on the right in Fig. 1b is a schematic of the molecular arrangement. The unit cell size was estimated to be 10 Å × 18 Å, which is in good agreement with that of previously reported DBBA monolayers.^32^ This molecular arrangement is expected to be ideal for long and anisotropic GNR growth because aligned DBBAs and the orientation of the Br atoms along the 〈110〉 direction would lead to efficient linear polymer chain formation.

**Fig. 2:**
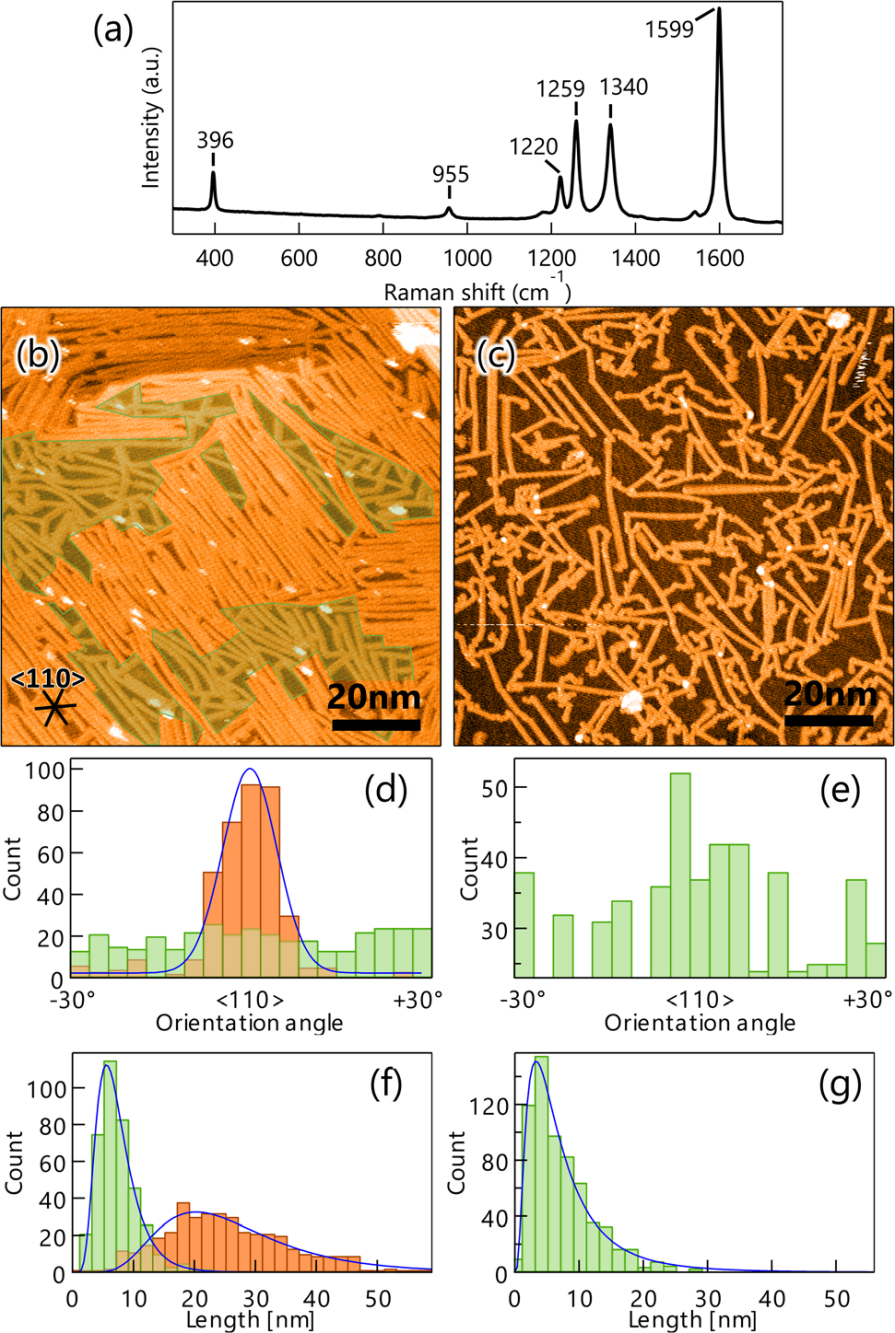
(a) Raman spectrum (532 nm) of graphene nanoribbons (GNRs) on Au(111) measured in the atmosphere. The peaks at 396 cm^−1^ and 955 cm^−1^ are characteristic of 7-aGNR. STM images of GNR on Au(111) synthesized by heating well-ordered 10,10′-dibromo-9,9′-bianthracenes monolayer (*V*_S_ = 1.0 V, *I*_T_ = 50 pA) (b) and the CVD method (*V*_S_ = 2.0 V, *I*_T_ = 100 pA) (c). The inserted lines represent the 〈110〉 direction of the substrate. The green shaded areas are GNR low-density areas. Histograms of orientation (d) and length (f) of GNR synthesized by the monolayer heating method on Au(111). Orange and green bars represent counts in high- and low-density areas with fittings shown by blue lines. Orientation histogram (e) and length histogram (g) of GNRs synthesized by the CVD method on Au(111) for comparison. The orientation histogram is divided and superposed every 60° owing to the sixfold symmetry of Au(111).

Next, the DBBA monolayer was polymerized *via* the Ullman coupling at 200 °C, and GNR was synthesized following the cyclodehydrogenation reaction at 400 °C. A typical Raman spectrum of the GNRs on Au(111) is shown in Fig. 2a. Peaks at 396 cm^−1^ and 955 cm^−1^ corresponding to 7-aGNR are clearly observed.^33^ We also carried out several Raman measurements at several different locations on the sample to confirm the uniform synthesis and found that Raman peaks at similar positions with similar intensities were observed over the surface (see Fig. S2a in ESI†). The Results indicate that the 7-aGNRs with a constant density form over the surface. Fig. 2b shows a representative STM image after GNR growth. The formation of high- and low-density (green-shaded parts in Fig. 2b) GNR domains can be observed over the surface. The average size of the high-density domain is 45 ± 10 nm in parallel to the GNRs and 42 ± 15 nm in perpendicular to the GNRs, with an area of 1.8 ± 0.9 × 10^3^ nm^2^, which accounts for 53 ± 9% of the surface (the maximum values were 63 nm in parallel to the GNRs, 62 nm in perpendicular to the GNRs, and an area of 3.9 × 10^3^ nm^2^, which accounts for 61%). Importantly, the STM analysis found that the well-ordered DBBA molecules were hardly desorbed from the surface by the heating process and efficiently converted into GNRs. When 1 ML DBBAs polymerize, the molecular distance of DBBAs in the direction Br–Br axis would be shortened to ∼50%, and the resultant produced GNR coverage should become ∼49% (see Fig. S3 and Table S1 in ESI†). The STM analysis found that the overall GNR coverage of ∼52%, which is in good agreement with that when 1 ML DBBA molecules are converted into GNRs without desorption. The results strongly indicate that the close-packed DBBA heating effectively suppressed the molecular desorption by the intermolecular interaction in the monolayer, expecting a contribution of long and aligned GNRs growth.

For comparison, GNR was synthesized by the CVD method. The resultant STM image shows the formation of short and randomly distributed GNRs over the surface (Fig. 2c), similar to those previously reported.^17^ Interestingly, the orientation and length distribution of GNRs prepared by the monolayer heating method were clearly different from those prepared by the diffusing precursor heating one. The monolayer heating method produced a high-density GNR domain consisting of long and oriented GNRs, and a low-density GNR domain consisting of short and randomly distributed GNRs. Unlike the monolayer heating method, the CVD method produced only short and randomly distributed GNRs with low-density dominance (Fig. 2c).

To obtain further insight into the differences, the orientation angle and growth length were quantified for both methods. Fig. 2d shows the orientation histogram of GNRs grown by the monolayer heating method. The histogram was divided and superposed every 60°, owing to the sixfold symmetry of Au(111). For the high-density domain, a single peak distribution in the 〈110〉 direction of Au(111) with a full width at half maximum (FWHM) of 12.2° was observed (orange bars in Fig. 2d), whereas the low-density domain was entirely isotropic (green bars in Fig. 2d). The length histogram also showed broad and sharp length distributions for the high- and low-density domains (orange and green bars in Fig. 2f) and mean GNR lengths of 20.4 and 5.6 nm, respectively. It is suggested that the intermolecular interaction plays an important role in the length of GNR growth.^34^ We also found that the lengths were shorter than the DBBA domain size before overheating, suggesting that both the GNR domains were created within the single DBBA domain.

The orientation and length histograms for the diffusing precursor heating method (only low-density GNR formation) are shown in Fig. 2e and g. Clearly, randomly distributed GNRs were formed with a short mean length of 3.6 nm, which is similar to that observed in the low-density domain of the well-ordered monolayer heating method. These results demonstrate that the well-ordered monolayer heating method enables longer and oriented GNR growth even though the short and randomly distributed GNR domain coexists. From these results, we can propose growth mechanisms for GNRs produced by the different growth methodologies. In the monolayer heating method, we observed the formation of high- and low-density GNR domains. For the high-density domain, the GNRs were oriented in the 〈110〉 direction, which was parallel to the DBBA molecular axis in the as-grown monolayer (Fig. 1b). The result indicates that the well-ordered DBBAs were polymerized while maintaining the molecular arrangement during heating. Because the DBBA molecules hardly desorbed and formed a closely packed monolayer, the molecular rotation would be effectively suppressed, thus, the individual molecular orientation was maintained even during heating. Because the molecular arrangement was maintained during the polymerization process, there were few opportunities for termination of the radical ends of the DBBA oligomers, such as through collision with other oligomers, resulting in the formation of longer and oriented GNRs along the 〈110〉 direction on Au(111). The GNR length would be dominated by the diffusion limitation of the grown oligomers. Simultaneously, the low-density area would be inevitably produced because the oligomer length was shorter than an integer multiple of the individual DBBAs due to the C–C covalent bond formation. Individual DBBA precursors in the low-density area would no longer be able to hold the defined orientation, thereby nucleating oligomers in a randomly oriented direction. As a result, the isotropic growth of the polymers in the spatially limited area would produce only short GNRs.

As for the diffusing precursor heating method, DBBAs are expected to diffuse freely on the surface and randomly polymerize. Since randomly oriented oligomers nucleate and collide, the formation of randomly distributed GNRs with short lengths would be predominant.

### GNR growth on Au(100) by well-ordered monolayer heating and CVD methods

**Fig. 3:**
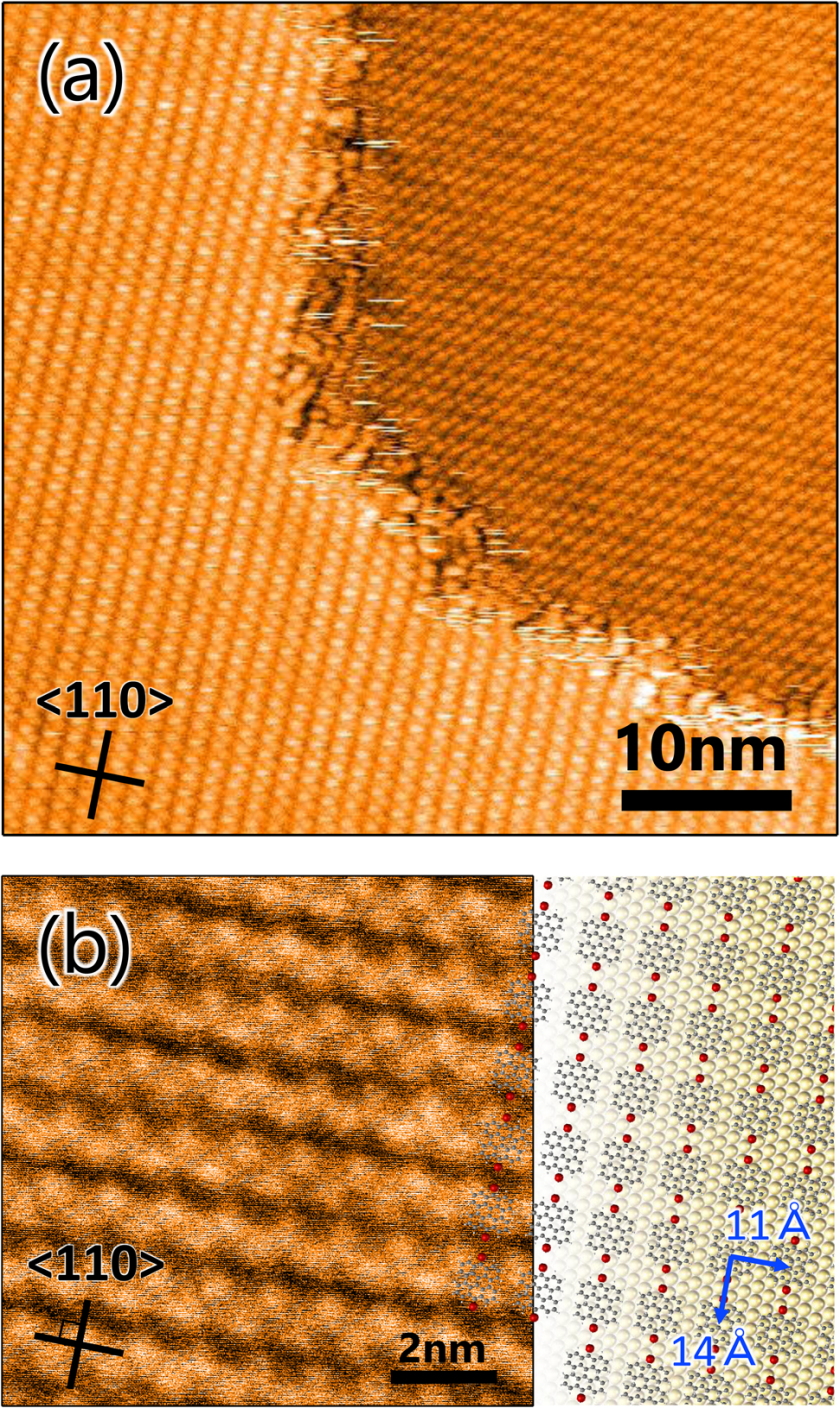
(a) STM image of 10,10′-dibromo-9,9′-bianthracenes monolayer on Au(100) (*V*_S_ = 2.0 V, *I*_T_ = 50 pA). (b) Enlarged STM image (*V*_S_ = 2.0 V, *I*_T_ = 100 pA) with molecular arrangement model expected from unit cell size (11 Å × 14 Å). The grey, red, and yellow balls represent carbon, bromine, and gold atoms, respectively. Inserted lines represent the 〈110〉 direction of the substrate.

It is very instructive to evaluate the effect of precursor molecule–surface interaction on GNR growth. The strength of the adsorbed molecular interaction on the surface depends on the crystalline surface plane, which thus affects the GNR growth. In this study, Au(100) was chosen for the comparative study because previous literature showed that organic molecules interact more strongly with the surface of Au(100) than that of Au(111).^35,36^ To verify the effect, a well-ordered DBBA monolayer was produced on Au(100) by sublimation, and STM analysis was carried out. The STM image of the as-grown DBBA monolayer on Au(100) (Fig. 3a) showed that a uniform well-ordered dense monolayer was formed, similar to that on Au(111). The domain sizes were also found to be larger than 100 nm, which is larger than that on Au(111). The zigzag protrusions in the enlarged image (Fig. 3b) corresponded to the up-tilted phenyl group in the twisted DBBA molecules. The consequent molecular arrangement model is depicted in Fig. 3b (right). The unit cell size was estimated to be 11 Å × 14 Å. The DBBA molecules were in a straight line and the Br atoms in each molecule were oriented in the 〈110〉 direction on Au(100), similar to that on Au(111) (Fig. 1a). Thus, the Br atoms of neighbouring precursors were much closer than Au(111). Furthermore, the DBBA monolayer formed on the Au(100)-hex reconstruction structure and the aligned DBBAs were parallel to the undulation of the reconstruction structure, as is the case with several other aromatic molecules.^37^ (see Fig. S4 in ESI†). This result strongly indicates that the GNRs on Au(100) grow along the surface undulation of the Au(100)-hex reconstruction.

**Fig. 4:**
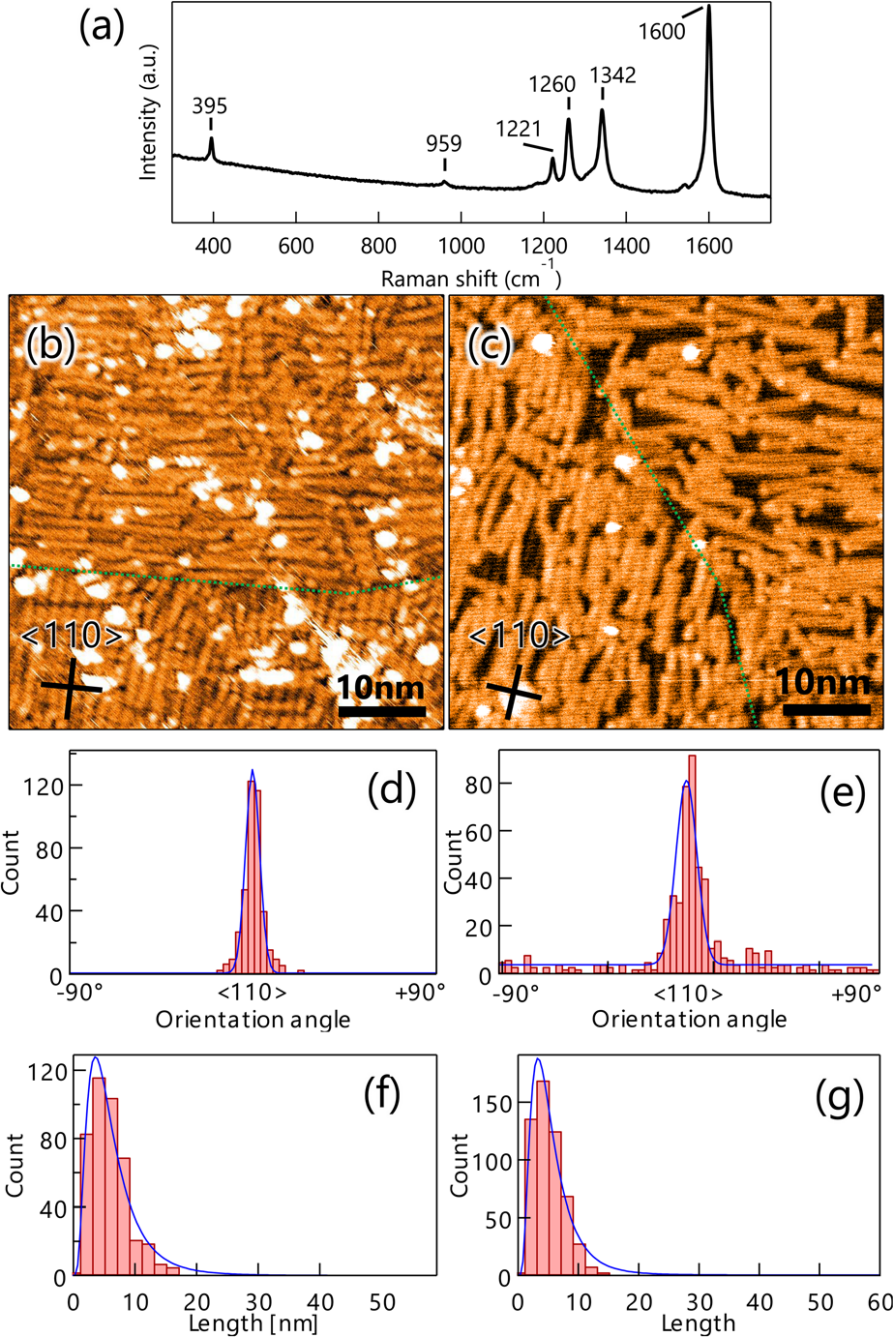
(a) Raman spectrum (532 nm) of graphene nanoribbons (GNRs) on Au(100) measured in the atmosphere. The peaks at 395 cm^−1^, 959 cm^−1^ are characteristic of 7-aGNR. STM images of GNR on Au(111) synthesized by heating well-ordered 10,10′-dibromo-9,9′-bianthracenes monolayer (*V*_S_ = 2.0 V, *I*_T_ = 100 pA) (b) and the CVD method (*V*_S_ = 2.5 V, *I*_T_ = 50 pA) (c). Green lines indicate domain boundaries. The inserted lines represent the 〈110〉 direction of the substrate. Histograms of orientation (d) and length (f) of GNR synthesized by the monolayer heating method on Au(100). Orientation histogram (e) and length histogram (g) of GNRs synthesized by the CVD method on Au(100) for comparison.

To produce GNRs on Au(100), the well-ordered DBBA monolayer on Au(100) was first polymerized at 200 °C and subsequently transformed into GNRs after heating at 400 °C. The Raman spectrum analysis of the GNR on Au(100) showed several peaks similar to those observed on Au(111) (Fig. 2a), confirming that the 7-aGNR formation on the Au(100) surface was similar to that on Au(111) (Fig. 4a). The peaks were observed with almost the same intensity at any location on the substrate, (see Fig. S2b in ESI†) such as GNRs on Au(111), indicating a constant density of the GNR distribution within the range of Raman spectroscopy measurements.

Fig. 4b shows a typical STM image of the produced GNRs on Au(100), where domains of GNRs oriented along the [11̄0] or [1̄10] directions with short lengths were found. The size of each domain was similar to that of DBBA domains before heating, suggesting that the orientation of DBBA before synthesis was preserved. The short GNRs were uniformly distributed on the entire surface even on the domain boundary, unlike the distribution pattern of GNRs synthesized on Au(111) (Fig. 2b). Furthermore, the GNR synthesis on Au(100) produced more residues than Au(111), suggesting that molecular residues produced under heating remain on Au(100) due to stronger interactions than Au(111). Noted that, as is the case of Au(111), DBBA molecules are hardly desorbed from the surface under the heating, forming GNR structure (see Fig. S3 and Table S1 in ESI†). We also synthesized GNRs on the surface of Au(100) by the CVD method to compare with the result of the monolayer heating method. STM analysis showed that GNRs grew on Au(100) with a slightly lower density than the monolayer heating method, but anisotropic growth was observed (Fig. 4c). The growth orientation is the same as that synthesized by the monolayer heating method, and is also consistent with the undulation of reconstructed Au(100) observed between the GNRs.

Distribution analyses of the length and orientation of the GNRs produced by both growth methods were carried out in a similar method as that of Au(111). The orientation histograms of GNRs on Au(100) produced by the monolayer heating method showed sharp peaks in the 〈110〉 direction with an FWHM of 4.8° and few randomly oriented GNRs (Fig. 4d). The GNRs produced by the CVD method also exhibited a slightly broader FWHM distribution of 6.9°, with a few randomly oriented GNRs (Fig. 4e). The mean GNR lengths for the monolayer heating and CVD methods were also estimated from the length histogram (Fig. 4f and g) to be 3.2 and 3.6 nm, respectively, showing that there was no difference in length between the two growth methods.

Taken together, the results showed that the GNRs on Au(100) grow along the surface undulation of the Au(100)-hex reconstruction for both growth methods, thereby being more anisotropic growth than that on Au(111). In contrast to the results on Au(111), the monolayer heating method showed few effects on the length and orientation of the GNR growth on Au(100). These results indicate that the observed anisotropic and short GNR growth for both methods is attributed to stronger adsorption of the precursors on anisotropic reconstructed Au(100) than on Au(111). According to previous studies, density functional theory calculation showed that aromatic benzene is adsorbed about 5% more strongly on Au(100) than on Au(111) (see Table S2 in ESI†).^35,36^ Furthermore, since the adsorption energy increases with the number of π-electrons of a molecule,^38^ the DBBA which has more π-electrons than benzene, should have a large difference in adsorption energy between Au(111) and Au(100).

### GNR formation model on Au(111) and Au(100) from monolayer heating method

**Fig. 5:**
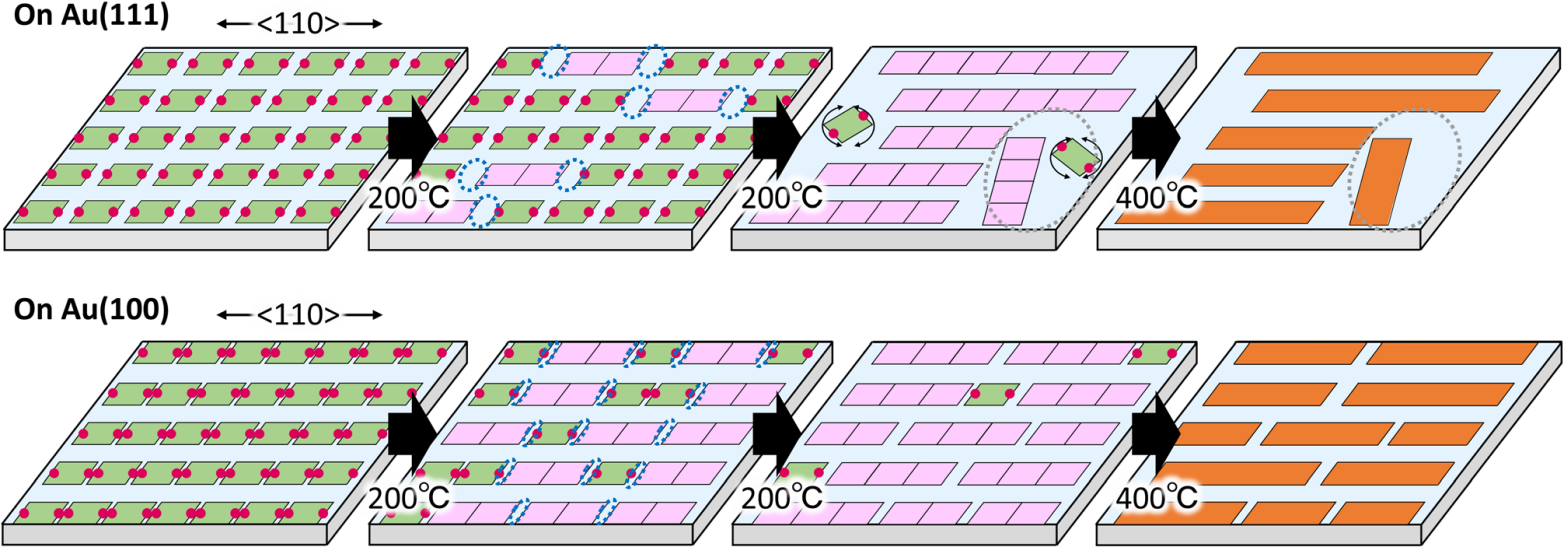
Schematic images of polymerization of 10,10′-dibromo-9,9′-bianthracenes (DBBA) on Au(111) and Au(100). Green, pink, and orange squares represent DBBA, DBBA oligomer, and graphene nanoribbons, respectively, and red balls represent Br atoms. Blue and grey dotted circles represent unfilled spaces and low-density area, respectively, created by DBBA polymerization.

Based on the above results, we discuss the difference in the GNR formation on Au(111) and Au(100). The difference is mainly ascribed to the anisotropic and strong interaction of the precursor on Au(100) compared to Au(111). Fig. 5 shows the proposed growth model of GNR formation on Au(111) (upper images) and Au(100) (lower images) by the monolayer heating method.

Sublimation of DBBAs on Au(111) and Au(100) produced well-ordered close-packed DBBA monolayers oriented with the molecular axes parallel to the 〈110〉 direction. The dense monolayer can suppress the molecular desorption from the surface under the heating because of intermolecular interaction in the monolayer. The situation can reduce the random diffusion and rotation of the precursors on the surface during the heating, contributing to aligned GNR growth. In particular, it is believed that the direction of the precursor strongly influences the growth direction of GNR during the oligomer nucleation.^39^ Therefore, heating the DBBA monolayer at 200 °C induced polymerization along the 〈110〉 direction, reflecting the molecular arrangement, and the consequent oligomer pieces would be nucleated (pink squares). The produced oligomers were shorter than an integer multiple of the individual precursors due to the formation of C–C covalent bonds between the DBBAs by debromination, and hence unfilled spaces would gradually be created along the 〈110〉 direction.

Because of the weaker interaction of the DBBAs and oligomers with Au(111), long-range, anisotropic diffusion of the precursors and oligomer pieces would be possible, resulting in the growth of longer and oriented polymers along the 〈110〉 direction. In addition, the diffusion of DBBA would be restricted to the 〈110〉 direction because the DBBA is closely packed in the 〈112〉 direction. The restriction would also contribute to long GNR growth, as is the case that GNR synthesis used monoatomic steps on metal surfaces in which the diffusion is restricted between adjacent terraces.^26–28^ In contrast, further growth would be suppressed by the decrease in diffusion of the grown oligomers. Simultaneously, unfilled spaces would grow in the plane direction, creating the spatially confined low-density area. Because the oligomers and precursors within the area could no longer retain the molecular arrangement, only short and randomly distributed polymers would grow in the area, similar to the GNR synthesis by diffusing precursor heating. Eventually, the resultant GNR formation on Au(111) by further heating produced two domain types: that composed of longer and densely oriented GNRs, and that composed of shorter and sparsely disordered ones, as shown in Fig. 2b.

In the case of Au(100), the intermolecular distance in the Br–Br axis direction during polymerization was closer than that on Au(111), leading to an increase in the oligomer nucleation frequency. The nucleation frequency was also increased by strong interaction between DBBA and Au(100). Furthermore, the strong interaction would reduce the diffusion length of the oligomers and precursors compared to Au(111). The diffusion differences would suppress polymer growth and unfilled plane space in the monolayer, resulting in only short and dense oriented GNRs (Fig. 4b).

## Conclusion

We studied GNR growth from the as-grown well-ordered dense DBBA monolayer on Au crystalline surfaces and first demonstrated the effectiveness of the growth technique by comparing it with the CVD method. STM analysis showed that DBBA molecules densely self-assembled on both Au(111) and Au(100) surfaces at RT, where the DBBAs aligned and adjusted the Br atoms in each molecule along the 〈110〉 direction. Under the heating, the dense monolayer can suppress the molecular desorption from the surface and can reduce the random diffusion and rotation of the precursors, contributing to aligned GNR growth. In the case of Au(111), subsequent heating of the monolayer induced polymerization along the 〈110〉 direction maintaining the molecular arrangement, and longer and anisotropic GNR growth was achieved compared to the CVD method. In the case of Au(100), further anisotropic GNR growths were observed for both growth methods, but the monolayer heating method showed few effects on the length and orientation of the GNR growth owing to the reduced diffusion of DBBAs and oligomers caused by their stronger interaction with Au(100) compared to Au(111). The results indicate that the strength between the adsorbate and Au surface also plays a significant role in longer and anisotropic GNR growth from the monolayer heating method. These findings provide deep insight into the design of longer and oriented GNR synthesis from the well-ordered precursor monolayer and serve in the development of GNR-based FETs.

## Conflicts of interest

There are no conflicts to declare.

## Supplementary Material

RA-013-D2RA07570A-s001
