# Supplementary material for: Long and oriented graphene nanoribbon synthesis from well-ordered 10,10′-dibromo-9,9′-bianthracene monolayer on crystalline Au surfaces
Source: RSC Adv. 2023 May 9;13(21):14089–96. doi: 10.1039/d2ra07570a (PMC10167794; doi:10.1039/d2ra07570a)
Supplement: RA-013-D2RA07570A-s001 [file RA-013-D2RA07570A-s001.pdf]

Electronic Supplementary Material (ESI) for RSC Advances.
This journal is © The Royal Society of Chemistry 2023

Supplementary Information

Long and Oriented Graphene Nanoribbon Synthesis from Well-ordered 10,10’-Dibromo-9,9’-Bianthracene Monolayer on Crystalline Au Surfaces

Masahiro Yano[a], Satoshi Yasuda,[a] Katsuyuki Fukutani[a,b] and Hidehito Asaoka*[a]*

[a] Research Group for Surface and Interface Science, Advanced Science Research Center, Japan Atomic Energy Agency, 2-4 Shirakata, Tokai, Ibaraki, 319-1195, Japan

[b] Institute of Industrial Science, The University of Tokyo, 4-6-1 Komaba, Meguro-ku, Tokyo 153-8505, Japan

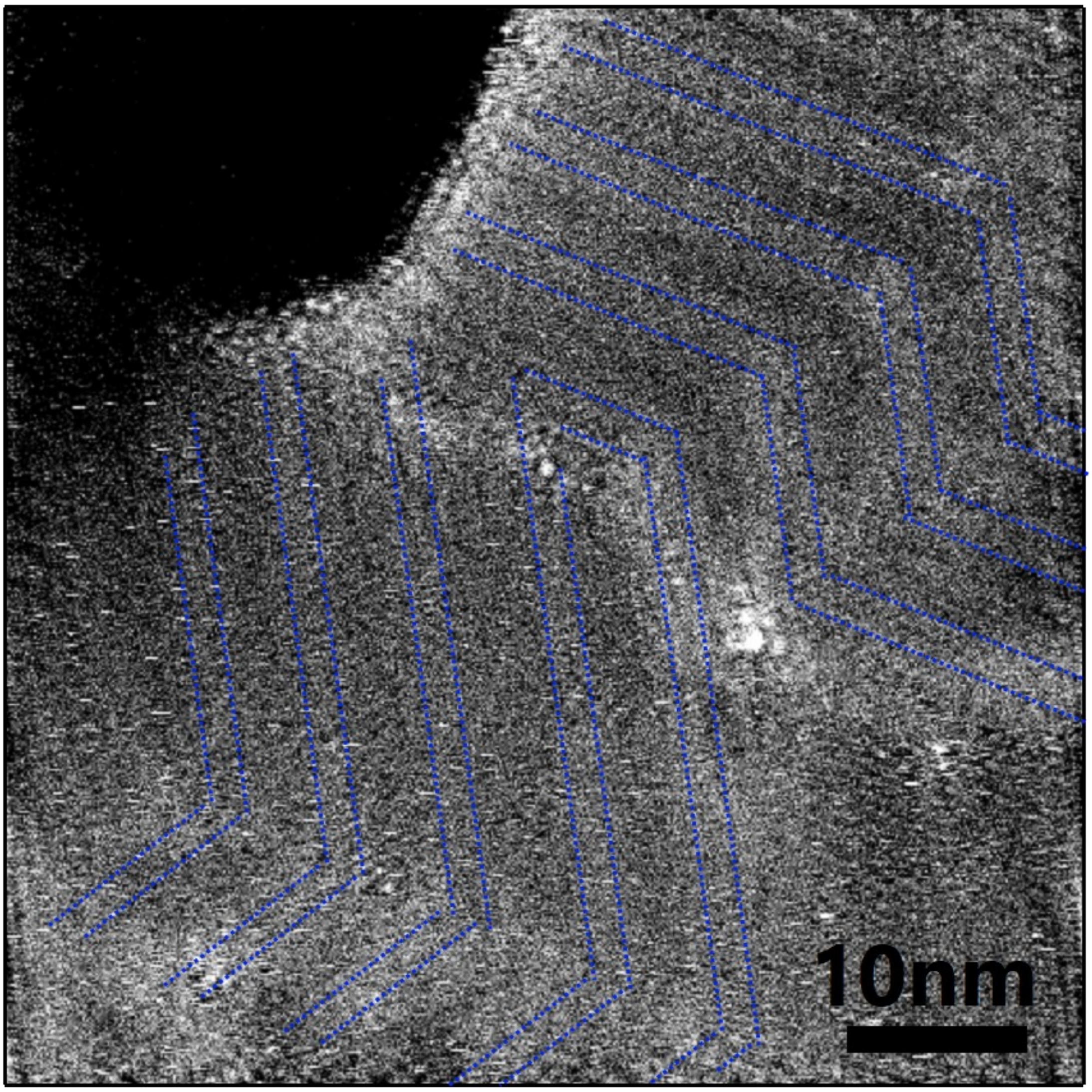

Figure S1. Processed image of Figure 1a. The contrast caused by DBBAs was removed by Fourier transform filtering to highlight the buried Au(111)-herringbone reconstruction structure. Blue lines represent the ridges of herringbone reconstruction.

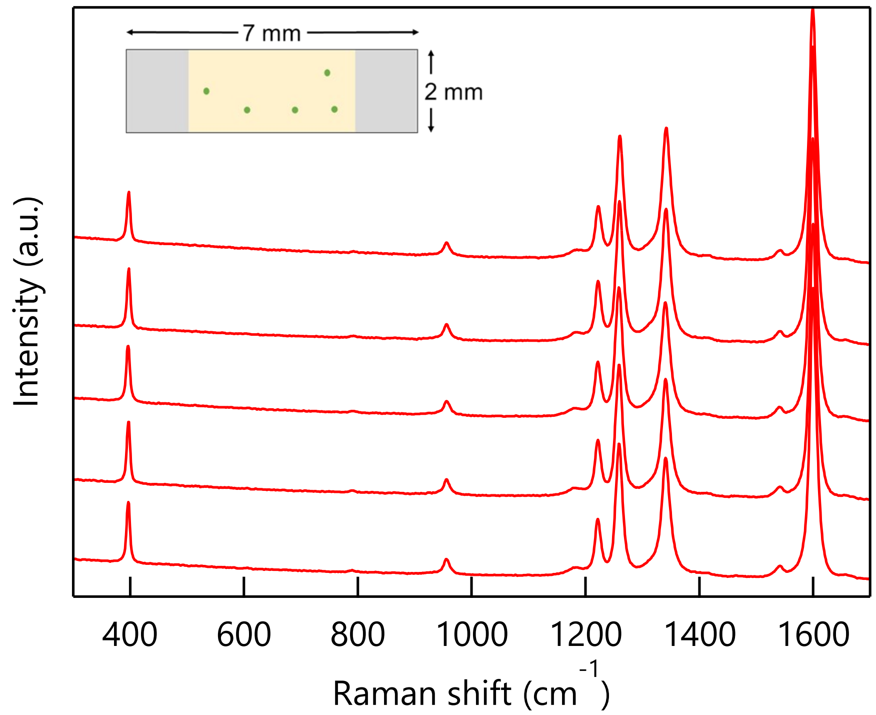

(a)

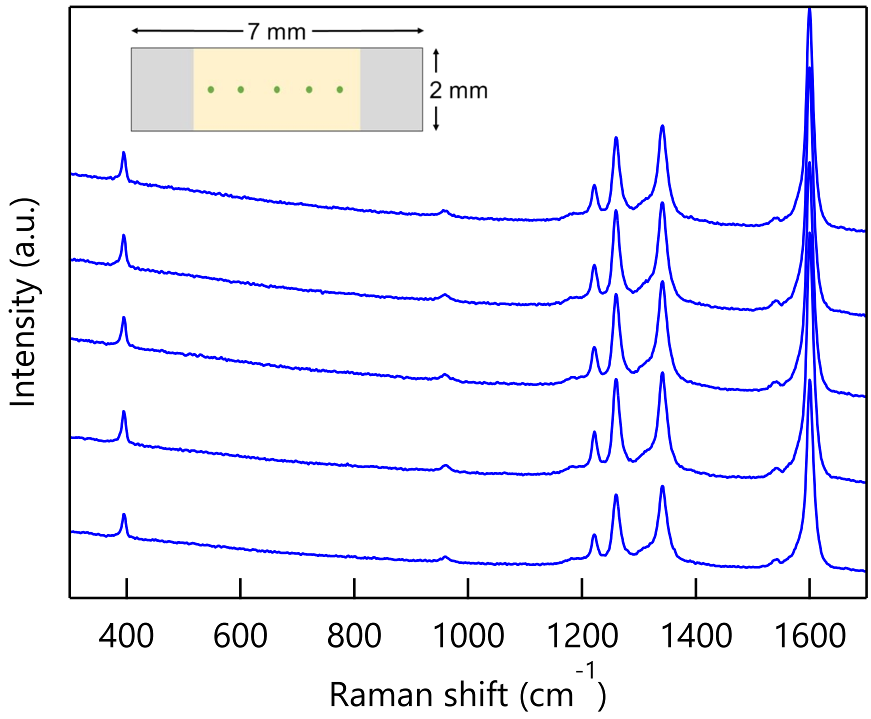

(b)

Figure S2. Raman spectra of GNR synthesized by the monolayer heating method on Au(111) (a) and Au(100) (b). The green dots in the inserted images show the positions where Raman spectroscopy measurements were performed on each Au substrate. The grey areas represent the positions where components were attached to hold the substrates.

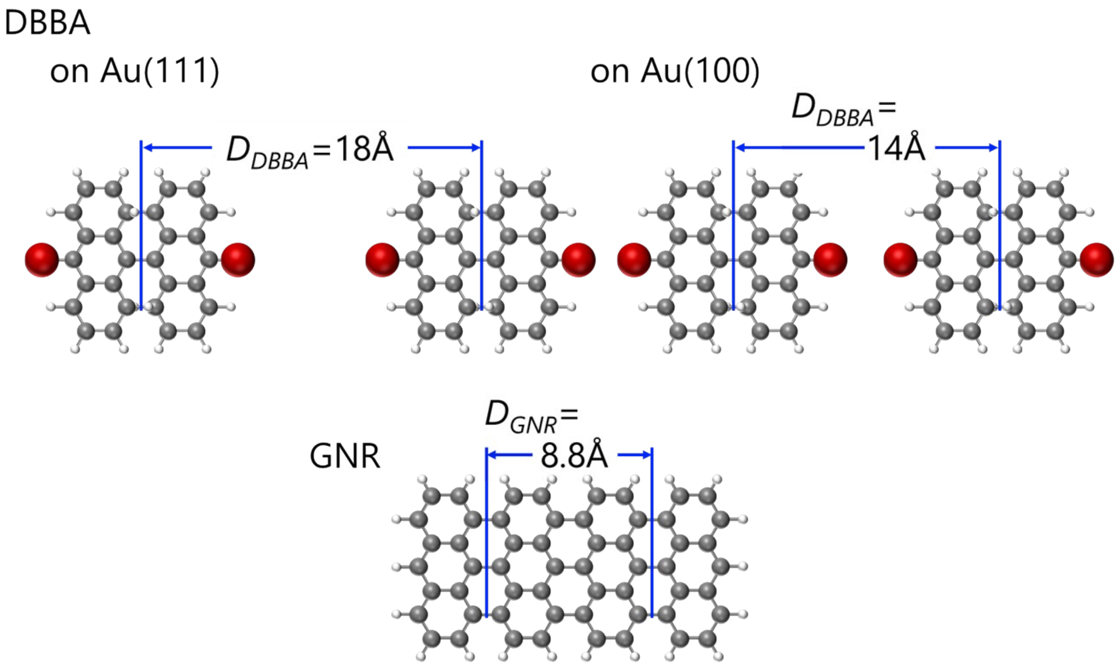

Figure S3. Schematic images of intermolecular distance before and after GNR synthesis. The lattice constant of GNR is based on the report by Q. Wang *et al.*.[1]

Table S1. Change in intermolecular distance and coverage before and after GNR synthesis.

	D_{DBBA} (Å)	GNR coverage (%) Calculation (D_{GNR}/D_{DBBA})	STM result
on Au(111)	18	49	52±1
on Au(100)	14	63	67±2

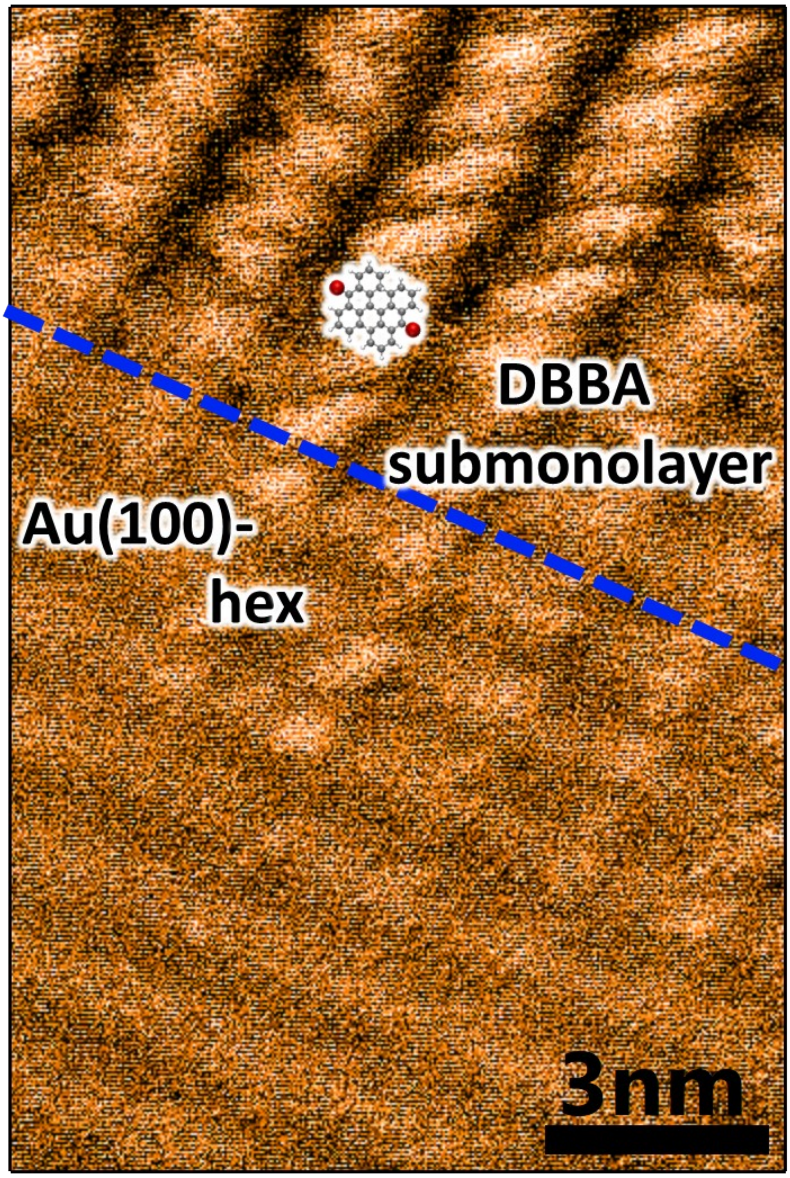

Figure S4. STM image around well-ordered DBBA submonolayer and Au(100)-hex boundary (V_S = 2.0 V, I_T = 100 pA). The observed DBBA supply for submonolayer fabrication is 65% of that for monolayer fabrication. Inset molecular image shows the adsorption position of DBBA molecules.

Table S2. The adsorption energy of benzene on Au(111) and Au(100) by theoretical calculations. PBE, RPBE, and RevPBE are DFT calculations using Perdew-Burke-Ernezerhof functionals. D3 and 3D(ABC) mean empirical dispersion correction. BJ means employing Becke-Johnson exchange potential.

[2] W. reckien *et al.*	E_{ads}(kJ/mol)	
	benzene/Au(111)	benzene/Au(100)
PBE-D3	-83	-87
PBE-D3(BJ)	-84	-89
PRBE-D3	-84	-85
RevPBE-D3	-105	-103
RevPBE-(BJ)	-121	-123
PBE-D3(ABC)	-70	-74
**[3] Y. Jiang *et al.***	E_{ad}(kJ/mol)	
	benzene/Au(111)	benzene/Au(100)
PBE+vdW^{sruf}	-72.4	-78.2
PBE+MBD	-54.0	-62.7
PBE	0.96	-8.68
MBD	-55.0	-54.0

References

[1] Wang *et al.*, J. Phys. Chem. C, 2021, **125**, 6034–6042

[2] W. Reckien *et al.,* J. Org. Chem., 2014, **10**, 1775–1784.

[3] Y. Jiang *et al.*, Sci Rep, 2016, **6**, 39529.
